# Perceived Burdensomeness, Thwarted Belongingness, and Social Exclusion in Transgender Women: Psychometric Properties of the Interpersonal Needs Questionnaire

**DOI:** 10.3389/fpsyg.2022.787809

**Published:** 2022-02-10

**Authors:** Yujie Liu, Rongxi Wang, Ruijie Chang, Huwen Wang, Lulu Xu, Chen Xu, Xiaoyue Yu, Shangbin Liu, Hui Chen, Yingjie Chen, Lian Jin, Ying Wang, Yong Cai

**Affiliations:** ^1^School of Public Health, Shanghai Jiao Tong University School of Medicine, Shanghai, China; ^2^Jockey Club School of Public Health and Primary Care, The Chinese University of Hong Kong, Hong Kong SAR, Hong Kong, China; ^3^Ban Song Yuan Road Community Health Service Centre, Shanghai, China

**Keywords:** Interpersonal Needs Questionnaire, transgender women, psychometric property, suicide, social exclusion

## Abstract

Transgender women (TGW) experience serious psychiatric problems and high suicide rates. According to the interpersonal theory of suicide, thwarted belongingness and perceived burdensomeness play major roles in suicidality and can be measured by the Interpersonal Needs Questionnaire (INQ). However, no study has validated the use of the INQ in TGW. This study aimed to examine the psychometric properties of the INQ among TGW. We recruited 198 TGW (mean age 38.47 years) from Shenyang, China, using snowball sampling. The construct validity of the INQ was assessed through factor analysis, and convergent and divergent validity were examined through a structural equation model with other psychosocial factors. The construct validation analysis supported a three-factor model (perceived burdensomeness, thwarted belongingness, and social exclusion) with satisfactory fit indices: *χ*^2^/df = 1.54, RMSEA = 0.052, CFI = 0.931, TLI = 0.916, SRMR = 0.053. The thwarted belongingness was significantly associated with self-esteem and social support, and the social exclusion was significantly associated with loneliness, depression, entrapment, and defeat, suggesting satisfactory convergent and divergent validity for the three-factor model. The present findings indicate that for TGW, high social exclusion is important in assessing perceived interpersonal needs, while the notable deviation from previous two-factor model warrants further study.

## Introduction

Transgender women (TGW) are those who were born with a male biological sex but have a female psychological sex and self-perception, regardless of medical interventions, such as gender reassignment surgery and hormone injections (Poteat et al., 2015; Becasen et al., 2019; Shi and Cai, 2020). A meta-analysis in 2015 showed that, considering only those seeking gender affirmation-related treatment at specialty clinics, transgender people represented about 4.6 per 100,000 of the total population globally, with 6.8 per 100,000 TGW and 2.6 per 100,000 transgender men. If all people questioning their gender were considered transgender, 0.5%~1.3% of the global population are TGW and 0.4% ~ 1.2% are transgender men (Winter et al., 2016). The TGW population suffers from widespread discrimination, especially gender-related discrimination at 47.5% (Becasen et al., 2019). Meanwhile, TGW experience serious mental health problems. A study reported that the depression rate of TGW was as high as 64% (Center for Epidemiologic Studies Depression Scale, CESD scale score ≥16; Nemoto et al., 2014), four times higher than the general population (Hyde et al., 2013). Another study in China reported approximately half the sample experienced higher levels of loneliness (51.1%) or was clinically depressed (49.1%; Shi and Cai, 2020). Furthermore, suicide rates are also high among TGW, with a review showing that suicide attempts in this population were 24.8% (95% CI: 18.0–33.2%; Becasen et al., 2019). In a US study, 41% of TGW reported having attempted suicide, compared with a frequency of 1.6% in the general population (Winter et al., 2016).

Death by suicide causes great pain to those close to them. The mechanisms of suicide have been studied for many years, and several theories have been proposed to explain it. The Interpersonal Theory of Suicide suggested that suicide occurs by the simultaneous presence of suicidal ideation and acquired capacity of self-harm and suicidal ideation results from unmet interpersonal needs (Joiner, 2005; Van Orden et al., 2008). According to the theory, various indices of social isolation, such as loneliness and low social support, are associated with suicide because they indicate that the need to belong has been thwarted—thwarted belongingness. Family dissension and unemployment are associated with suicide since they are probably to induce perception of burdensomeness on others—perceived burdensomeness. Unmet interpersonal needs may in turn intensify low self-esteem, loneliness, lack of social support, and depression (Van Orden et al., 2010, 2012; Park and Kim, 2019).

Thwarted belongingness and perceived burdensomeness are proximal and sufficient causes of suicidal thoughts and can be measured by the Interpersonal Needs Questionnaire (INQ) developed by Joiner and Kimberly (Joiner, 2005; Van Orden et al., 2008). INQ has been validated among college students (Becker et al., 2020; Loas et al., 2021), young male adults (Teo et al., 2018), community-dwelling older persons (Shim et al., 2021), current firefighters (Chu et al., 2016), clinically depressed and suicidal youth (Hunt et al., 2020) and military veterans (O’Connor et al., 2017), and translated to different languages (Podlogar et al., 2017; Silva et al., 2018; Park and Kim, 2019; Iliceto et al., 2021; Lai and Boag, 2021). While the two-factor structure—thwarted belongingness and perceived burdensomeness—showed reliable and acceptable model fit to college students, older people, and firefighters, studies among clinically depressed and suicidal youth (Hunt et al., 2020) and migrant workers (Wang et al., 2021a) validated a better model fit for three dimensions of the INQ. These particular groups like TGW and suicidal youth shared similar experiences of loneliness and as Quintin A. Hunt suggested, loneliness is not accurately measured as through the TB subscale (Hunt et al., 2020). Meanwhile, TGW reported more experiences of family rejection, discriminatory comments from family members, alienation from friends, unemployment, beatings, rape, etc. (Poteat et al., 2017). Given the traditional conservative culture in China, transgender people are excluded from the mainstream of society which indicated that the belonging need for them may be different from other population. It is reasonable to question that whether a two-factor structure of INQ remains unchanged in TGW. Furthermore, early identification of suicidal ideation before suicide attempt can help prevent suicide considering the high suicide rate among this population.

Although the Chinese version of the INQ has been widely used to measure interpersonal needs and predict suicidal ideation, its psychometric properties remained unclear in TGW population. The purpose of the present study is twofold: to explore the dimension structure of INQ through exploratory and confirmatory factor analysis (CFA) and to assess the convergent and divergent validity of INQ among TGW using structure equation model, determining whether the INQ is a reliable and valid assessment tool among TGW in China.

## Materials and Methods

### Participants

The cross-sectional survey of TGW in Shenyang, China was conducted from April to July 2017. A snowball sampling method was used for recruitment with the help of a non-governmental organization dedicated to improving the physiological and psychological health of TGW. An initial set of five eligible participants completed the survey, then they were asked to recommend others to be recruited into the study. The participants forwarded the questionnaire survey in this way until they could not identify others who meet the inclusion criteria. The inclusion criteria were: (1) aged 18 years or older; (2) had male biological sex; (3) had female psychological sex; (4) had the ability to understand the questionnaire; and (5) informed about the study procedure and signed written informed consent.

All participants were informed about the assurance of anonymity during the survey, the right to withdraw at any time, and that there would be no consequence of refusing the survey. A total of 198 TGW were finally recruited in the study. Participants’ ages ranged from 18 to 62 years, with a mean age of 33.47 years.

### Measures

#### Chinese Version of the INQ-15

The 15-item INQ, adapted from the original 25-item INQ, was designed to measure suicide-related interpersonal needs (Van Orden et al., 2012). The two-factor measurement model of the INQ-15 has been validated in several previous research, which measures two dimensions: perceived burdensomeness and thwarted belongingness (Amini-Tehrani et al., 2021; Iliceto et al., 2021). In the INQ-15, each item is assessed on a 7-point Likert scale from “not true for me at all” to “very true for me.” Total score of the INQ-15 is between 0 and 105, with higher scores indicate severer unmet interpersonal needs. The Chinese version of INQ-15, which was translated by Chen, demonstrated good internal consistency among college students, with Cronbach’s alpha values ranging from 0.72 to 0.93 for perceived burdensomeness and 0.78 to 0.91 for thwarted belongingness (Li et al., 2015; Lai and Boag, 2021).

#### Chinese Version of the RSES

The Rosenberg Self-Esteem Scale (RSES) was developed by Rosenberg to measure the degree of self-esteem (Rosenberg, 1989). The scale consists of ten items that evaluate two dimensions of self-esteem: self-competence and self-liking. Total scores of the RSES range from 10 to 40, and higher scores indicate higher level of self-esteem. The Chinese version of the RSES was translated by Ji and exhibited adequate internal consistency in migrants and adolescents (Cronbach’s alpha = 0.81; Liang et al., 2020; Wang et al., 2021b).

#### Chinese Version of the 8-Item UCLA Loneliness Scale (ULS-8)

The 8-item ULS is a self-reported measure of perceived loneliness, which was refined from the original 20-item ULS by Hays (Hays and DiMatteo, 1987). In the ULS-8, respondents were asked about the frequency of possible negative experiences associated with social relationships (i.e., “I lack companionship”), ranging from 1 (“never”) to 4 (“always”). Higher scores of ULS-8 indicate greater level of perceived loneliness. The Chinese version of the ULS-8 was translated by Zhou and demonstrated good internal consistency in adolescents and older adults (Cronbach’s alpha = 0.831–0.878; Zhou et al., 2012; Xu et al., 2018).

#### Chinese Version of the 9-Item PHQ-9

The Patient Health Questionnaire (PHQ-9) is an instrument for assessing the severity of depressive symptoms, which was derived from the depression module from the self-administered mental health diagnostic instrument PHQ (Kroenke et al., 2001). The scale consists of nine 4-point Likert-type items from 0 (“not at all”) to 4 (“nearly every day”), with higher scores indicate severe depressive symptoms. The Chinese version of the PHQ-9 has shown good internal consistency among adolescents, with Cronbach’s alpha value between 0.84 and 0.86 (Tsai et al., 2014; Leung et al., 2020).

#### Chinese Version of the ES

The 16-item Entrapment Scale (ES) was developed by Gilbert and Allan to measure the extent an individual feels imprisoned or trapped by unbearable thoughts, feelings or circumstances (Gilbert and Allan, 1998). The scale consists of two dimensions: external entrapment (EE) and inner entrapment (IE). In the ES, each item is assessed on a 5-point Likert scale from 0 (“not at all like me”) to 4 (“extremely like me”), and higher scores indicate greater feeling of entrapment. The Chinese version of the ES has exhibited good internal consistency among college students (Cronbach’s alpha = 0.94 for EE, 0.93 for IE; Gong et al., 2019).

#### Chinese Version of the DS

The 16-item ES was developed by Gilbert and Allan to measure the sense of failed struggle and low social rank (Gilbert and Allan, 1998). The scale consists of two dimensions: decadence and low sense of achievement. In the Defeat Scale (DS), each item is assessed on a 5-point Likert scale from 0 (“never”) to 4 (“always”), and higher scores indicate greater feeling of entrapment. The Chinese version of the DS has exhibited good internal consistency among college students (Cronbach’s alpha = 0.933; Tang et al., 2019).

#### Chinese Version of the MSPSS

The multidimensional scale of perceived social support (MSPSS) is a 12-item self-reported measure of perceived social support from three sources: family, friends, and significant others. Respondents were asked to report their agreement with items (i.e., “My family really tries to help me”) on a 7-point Likert scale (Zimet et al., 1988). Total scores of the MSPSS are between 12 and 84, with higher scores indicate higher perceived social support. The Chinese version of the DS has demonstrated adequate internal consistency among college students (Cronbach’s alpha = 0.86 for family; 0.89 for friends; 0.86 for significant other; Zhang et al., 2018).

### Statistical Analysis

All analyses were performed using Mplus version 7.4 and R 4.0.0. Descriptive statistics were conducted for INQ items and psychosocial factors using R 4.0.0. Reliability of the INQ was assessed by internal consistency using Cronbach’s alpha coefficient, with a coefficient of greater than 0.7 indicates good internal consistency (Nunnally and Bernstein, 1994).

Exploratory factor analysis and CFA were conducted to examine the factor structure of the scale using Mplus version 7.4. Maximum likelihood estimation with robust standard errors with geomin oblique rotation was utilized because the majority of INQ items were non-normally distributed. Additionally, the INQ was analyzed *via* Item Response Theory (IRT) models to obtain more complex information about the psychometric properties of the individual assessment items. A series of fit indices were used to examine the goodness of fit of the model: chi-square (*χ*^2^), standardized root mean square residual (SRMR), comparative fit index (CFI), Tucker–Lewis index (TLI), and the root mean squared error of approximation (RMSEA). The assessment criteria for each index were: *χ*^2^/df < 3 (*p* > 0.05), SRMR<0.08, CFI > 0.9, TLI > 0.9, RMSEA <0.08 (McDonald and Ho, 2002).

The statistical power (*π*) of the CFA model was estimated by Monte Carlo simulation. In Monte Carlo simulation, a hypothesized population value for each model parameter was set identical to the established CFA model. The model was estimated based on a large number of randomly generated samples. Muthén and Muthén suggested that the following criteria should be meet to achieve a desirable level of *π* (0.80) with a certain sample size: (1) parameter and standard error bias <10%; (2) standard error bias for the parameter of interest <5%; and (3) coverage (the proportion of samples for which the 95% confidence interval contains the parameter value) between 0.91 and 0.98 (Muthén and Muthén, 2002).

A structural equation model with the six psychosocial variables and INQ was constructed using Mplus version 7.4. All the observed variables were regressed onto the latent factors of the INQ-15 in the model. The magnitude of the regression coefficients indicated whether dissimilar constructs of the scale could be distinguished by different observed variables.

### Ethics

The study procedures were carried out according to the Declaration of Helsinki. The Institutional Review Board of the School of Public Health, Shanghai Jiao Tong University School of Medicine approved this study. All participants were informed about the study, and all provided informed consent.

## Results

### Description of the INQ-15

Descriptive statistics (i.e., number, mean, standard error, standard deviation, variance, skewness, kurtosis, and range) and inter-correlations between the 15 items for the whole sample are presented in Table 1.

**Table 1:** Descriptive statistics and inter-correlations of the interpersonal needs questionnaire items.

	Item1	Item2	Item3	Item4	Item5	Item6	Item7	Item8	Item9	Item10	Item11	Item12	Item13	Item14	Item15
Item1	1.000	0.710^**^	0.468^**^	0.500^**^	0.441^**^	0.399^**^	0.152^*^	0.207^**^	0.229^**^	0.165^*^	0.286^**^	0.360^**^	0.145^*^	−0.182^*^	0.228^**^
Item2		1.000	0.432^**^	0.591^**^	0.470^**^	0.455^**^	0.178^*^	0.287^**^	0.213^**^	0.184^**^	0.400^**^	0.395^**^	0.203^**^	0.251^**^	0.199^**^
Item3			1.000	0.577^**^	0.511^**^	0.452^**^	0.236^**^	0.280^**^	0.297^**^	0.224^**^	0.409^**^	0.376^**^	0.179^*^	0.251^**^	0.204^**^
Item4				1.000	0.532^**^	0.550^**^	0.181^*^	0.302^**^	0.254^**^	0.160^*^	0.369^**^	0.479^**^	0.182^*^	0.238^**^	0.250^**^
Item5					1.000	0.553^**^	0.208^**^	0.203^**^	0.305^**^	0.211^**^	0.432^**^	0.436^**^	0.206^**^	0.225^**^	0.276^**^
Item6						1.000	0.256^**^	0.211^**^	0.309^**^	0.182^*^	0.387^**^	0.380^**^	0.202^**^	0.188^**^	0.206^**^
Item7							1.000	0.488^**^	0.265^**^	0.574^**^	0.219^**^	0.336^**^	0.458^**^	0.445^**^	0.315^**^
Item8								1.000	0.104	0.388^**^	0.296^**^	0.279^**^	0.399^**^	0.423^**^	0.391^**^
Item9									1.000	0.299^**^	0.441^**^	0.398^**^	0.314^**^	0.291^**^	0.219^**^
Item10										1.000	0.273^**^	0.328^**^	0.580^**^	0.539^**^	0.348^**^
Item11											1.000	0.553^**^	0.268^**^	0.266^**^	0.245^**^
Item12												1.000	0.283^**^	0.324^**^	0.332^**^
Item13													1.000	0.543^**^	0.427^**^
Item14														1.000	0.550^**^
Item15															1.000
*N*	198	198	198	198	198	198	198	198	198	198	198	198	198	198	198
Mean	2.26	2.29	1.79	1.95	1.88	1.84	1.84	4.63	2.70	5.51	2.59	2.57	5.25	4.90	4.52
SE	0.111	0.119	0.101	0.115	0.096	0.104	0.104	0.146	0.136	0.127	0.134	0.136	0.126	0.128	0.140
SD	1.561	1.672	1.427	1.619	1.350	1.467	1.467	2.048	1.914	1.790	1.880	1.921	1.776	1.804	1.971
Var	2.436	2.797	2.036	2.622	1.823	2.153	2.153	4.195	3.664	3.206	3.533	3.689	3.154	3.255	3.885
Skew	1.029	1.191	2.074	1.770	1.636	1.979	1.979	−0.384	0.822	−1.094	0.926	0.965	−0.855	−0.521	−0.308
Kurt	0.220	0.554	3.956	2.276	2.175	3.391	3.391	−1.048	−0.563	0.183	−0.284	−0.297	−0.114	−0.601	−0.978
Range	6	6	6	6	6	6	6	6	6	6	6	6	6	6	6

*SD, Standard deviation; SE, Standard error; Var, Variance; Skew, Skewness; Kurt, Kurtosis.*

* p < 0.05

** p < 0.01.

### Construct Validity

#### The Exploratory Factor Analysis

The results of EFA with 1–4 factors yielded three eigenvalues greater than one (Table 2). Both the three-factor model and the four-factor model displayed good model fits on *x*^2^/df, RMSEA, CFI, TLI, and SRMR. After examining factor structures of the two models, the three-factor solution had better indicators for each factor and was finally decided (Table 3). The pattern of the loadings for the model indicated that the first six items (1/2/3/4/5/6) loaded onto the “perceived burdensomeness” factor and item 7/8/10/13/14/15 loaded onto the “thwarted belongingness” factor. The remaining three items (9/11/12) loaded onto a third factor which was named “social exclusion.”

**Table 2:** Model fit indices for the Interpersonal Needs Questionnaire.

Factor	*χ* ^2^	df	Value of *p*	RMSEA	90%CI	CFI	TLI	SRMR	Eigenvalue
1	354.801	90	<0.005	0.122	0.109–0.135	0.612	0.547	0.119	4.964
2	174.735	89	<0.005	0.07	0.054–0.085	0.874	0.852	0.069	2.324
3	134.388	87	0.0008	0.052	0.034–0.069	0.931	0.916	0.053	1.169
4	134.124	85	0.0005	0.054	0.036–0.071	0.928	0.911	0.053	0.917

*χ*^2^, Chi-square; SRMR, Standardized root mean square residual; CFI, Comparative fit index; TLI, Tucker–Lewis index; RMSEA, Root mean squared error of approximation.

**Table 3:** Factor structure of the Interpersonal Needs Questionnaire.

	Three-factor model		
	PB	TB	SE
1.Better off	**0.765**	−0.159	0.145
2.Happier out me	**0.804**	−0.213	0.119
3.Burden to society	**0.642**	−0.267	0.369
4.Death as relief	**0.847**	−0.22	0.192
5.Rid of me	**0.722**	−0.231	0.418
6.Makes worse	**0.687**	−0.234	0.338
7.Others care	−0.162	**0.701**	−0.197
8.I belong	−0.324	**0.673**	0.002
9.Rarely interact	0.173	−0.209	**0.825**
10.Friends	−0.11	**0.742**	−0.22
11.Disconnected	0.446	−0.26	**0.737**
12.Outsider	0.496	−0.385	**0.578**
13.Turn to	−0.126	**0.731**	−0.248
14.Close to others	−0.209	**0.783**	−0.222
15.Daily interact	−0.289	**0.666**	−0.179

*PB, Perceived burdensomeness; TB, Thwarted belongingness; SE, Social exclusion; S.E., Standard error. Bold: significant at 5% level.*

#### The Confirmatory Factor Analysis

Estimated parameters for the three-factor model (standardized estimated factor loadings, standard error, covariances, and *R*^2^ values) are presented in Table 4. All items significantly loaded onto the anticipated latent factors revealed by the EFA, with *R*^2^ ranging from 0.195 to 0.640. The three factors (perceived burdensomeness, thwarted belongingness, and social exclusion) were distinct but closely correlated, with perceived burdensomeness and social exclusion positively correlated with each other (*r* = 0.641) while negatively correlated with thwarted belongingness (*r* = −0.338; *r* = −0.474).

**Table 4:** Estimated parameters for the three-factor model.

	Estimated	S.E.	*p*	*R* ^2^
**Perceived burdensomeness**				
1.Better off	0.686	0.065	<0.001	0.471
2.Happier out me	0.730	0.060	<0.001	0.533
3.Burden to society	0.614	0.084	<0.001	0.377
4.Death as relief	0.800	0.039	<0.001	0.640
5.Rid of me	0.694	0.061	<0.001	0.482
6.Makes worse	0.657	0.074	<0.001	0.432
**Thwarted belongingness**				
7.Others care	0.605	0.072	<0.001	0.366
8.I belong	0.578	0.063	<0.001	0.334
10.Friends	0.662	0.072	<0.001	0.438
13.Turn to	0.672	0.069	<0.001	0.452
14.Close to others	0.748	0.052	<0.001	0.560
15.Daily interact	0.615	0.059	<0.001	0.378
**Social exclusion**				
9.Rarely interact	0.442	0.082	<0.001	0.195
11.Disconnected	0.714	0.063	<0.001	0.510
12.Outsider	0.742	0.069	<0.001	0.551
**Covariances**				
SE WITH PB	0.641	0.075	<0.001	
SE WITH TB	−0.474	0.093	<0.001	
PB WITH TB	−0.338	0.083	<0.001	

*PB, Perceived burdensomeness; TB, Thwarted belongingness; SE, Social exclusion; S.E., Standard error*.

#### IRT Modeling and M2 Test

The results of M2 test indicated good model fit of the IRT modeling: RMSEA = 0.037 (90% CI = 0.009–0.065), M2 = 11.94 (*p* = 0.036).

#### Statistical Power Estimation

In Monte Carlo simulation, the parameter and standard error bias were 0.74% and − 2.58% for factor 1 and factor 2, −0.18% and − 1.85% for factor 1 and factor 3, and − 0.21% and − 4.22% for factor 2 and factor 3. All the parameter and standard error bias did not exceed 5%, and all the coverage remained above the minimum threshold of 0.91. Therefore, a sample size of 198 in this study was enough for a *π* of 0.80. Results of Monte Carlo simulation are presented in Supplementary Material.

### Reliability

Cronbach’s alpha values for the Chinese version of the INQ-15 were 0.848 for the total scale, 0.849 for perceived burdensomeness, 0.808 for thwarted belongingness, and 0.660 for social exclusion. The internal consistency was satisfactory for the total scale as well as the first two factors. Although social exclusion had a comparatively low Cronbach’s alpha value, it is acceptable for a subscale with three items (Rust and Golombok, 1999).

### Convergent and Divergent Validity

#### Description of Psychosocial Factors

**Figure 1:**
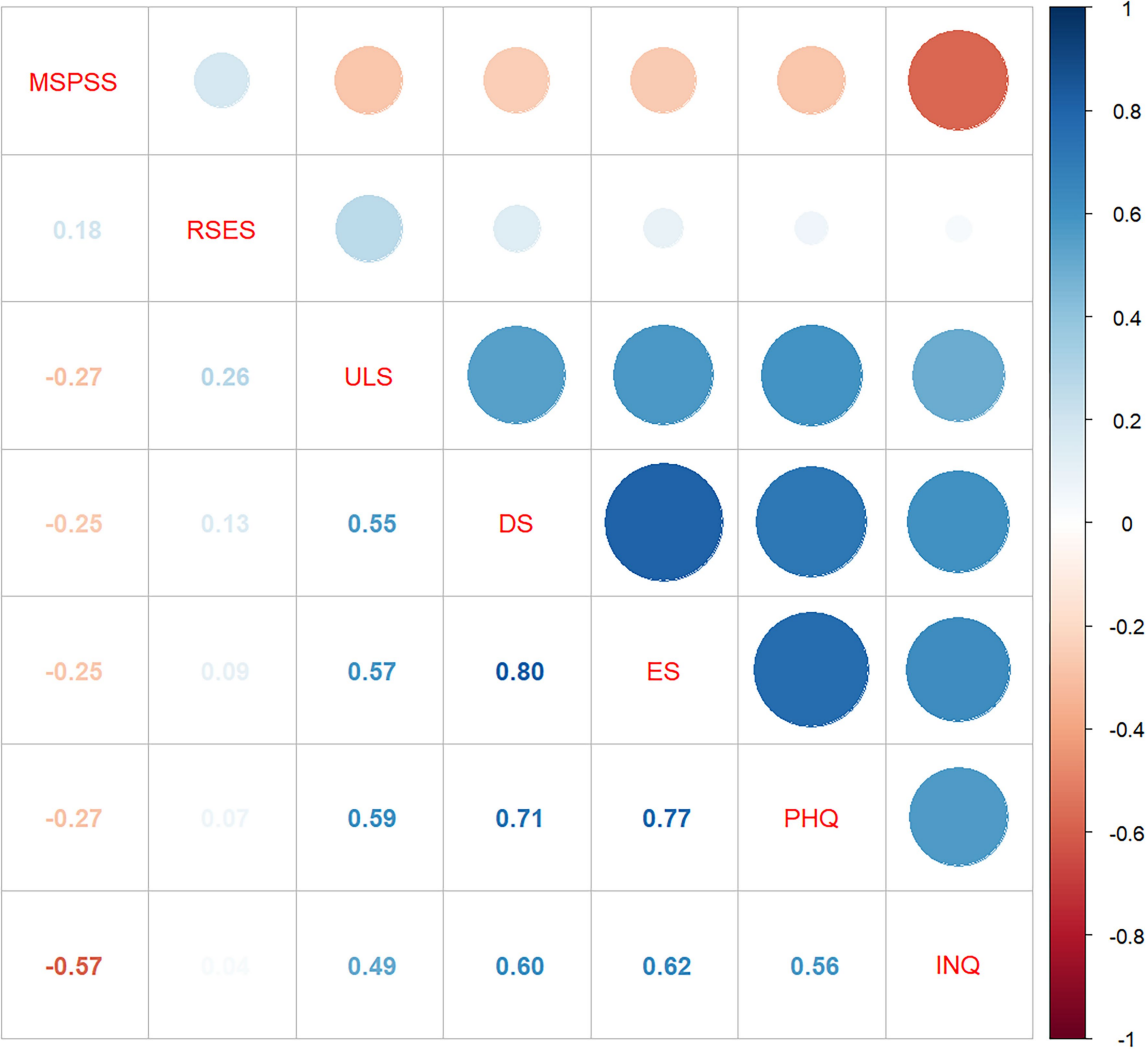
Inter-correlations among psychosocial factors.

Descriptive statistics (i.e., number, mean, standard error, standard deviation, variance, skewness, kurtosis, and range) of the psychosocial factors (i.e., perceived social support, loneliness, depression, self-esteem, entrapment, and defeat) for the whole sample are presented in Table 5. Figure 1 showed the inter-correlations among different psychosocial factors, and interpersonal needs is positively correlated with loneliness (*r* = 0.49), depression (*r* = 0.56), entrapment (*r* = 0.62), defeat (*r* = 0.60), and negatively correlated with perceived social support (*r* = −0.57).

**Table 5:** Descriptive statistics of the psychosocial factors.

	Mean	Median	SD	SE	Range	Skew	Kurtosis
INQ	37.85	36.00	14.797	1.052	63	0.442	−0.262
INQ_PB	12.02	10.00	6.887	0.489	36	1.472	2.384
INQ_TB	17.98	17.00	7.996	0.568	35	0.426	−0.206
INQ_SE	7.85	7.50	4.410	0.313	18	0.573	−0.571
Self_esteem	26.33	26.00	2.746	0.195	22	0.048	2.379
Loneliness	18.36	18.00	4.282	0.304	22	0.658	−0.030
Depression	6.55	5.00	5.699	0.405	27	1.250	1.856
Entrapment	15.44	10.00	15.578	1.107	63	0.952	−0.122
Defeat	17.53	14.00	12.138	0.863	53	1.064	0.454
Social_support	62.53	64.00	14.216	1.010	69	−0.716	0.199

*INQ, Interpersonal need; PB, Perceived burdensomeness; TB, Thwarted belongingness; SE, Social exclusion; SD, Standard deviation; SE, Standard error; Skew, Skewness; Kurt, Kurtosis*.

#### Convergent and Divergent Validity

**Figure 2:**
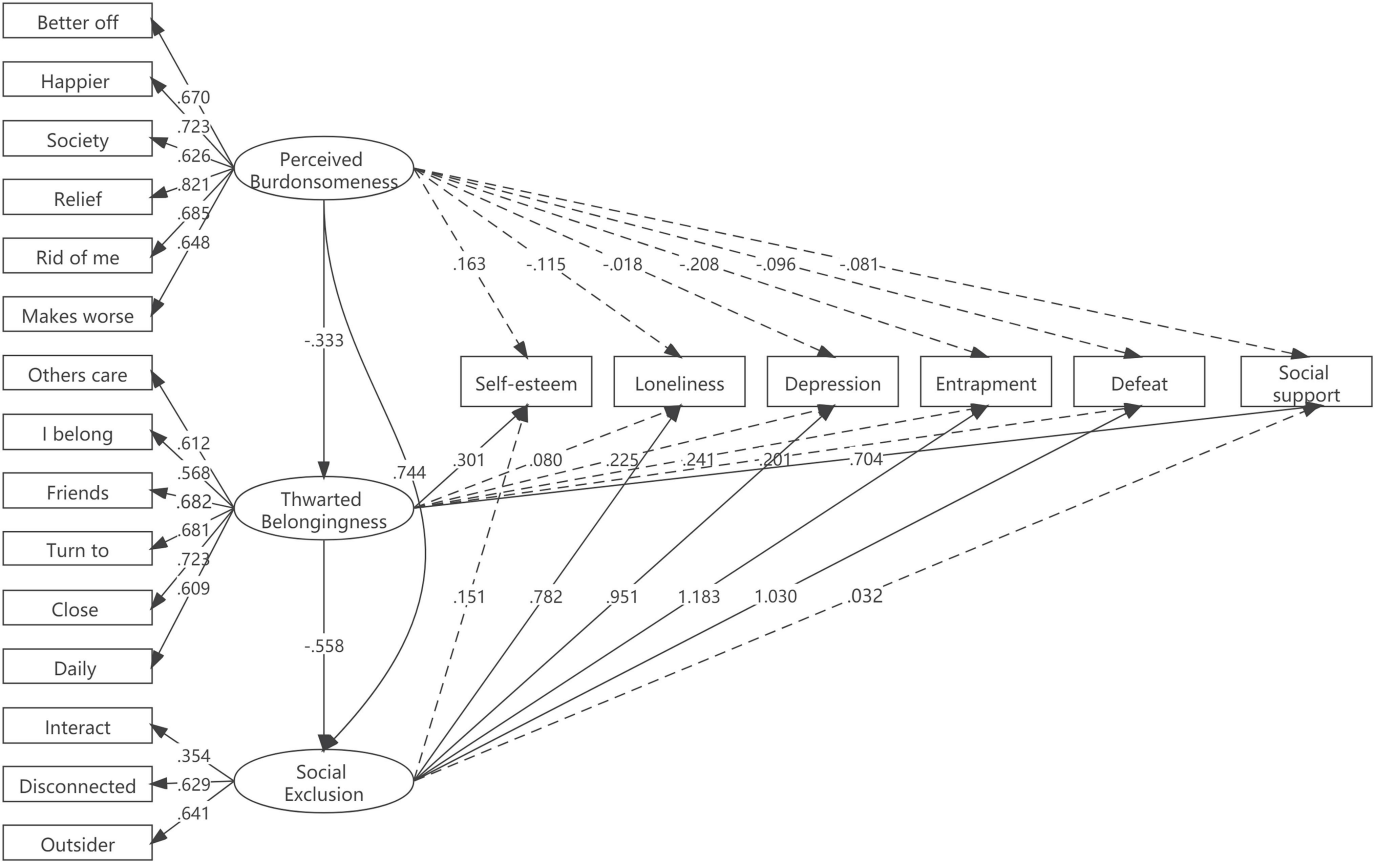
Structural equation model results examining convergent and divergent validity.

Figure 2 presented the results of the simultaneous regression of the six psychological factors on the three latent factors of perceived burdensomeness, thwarted belongingness, and social exclusion. The thwarted belongingness was associated with self-esteem and social support (*p* < 0.05); the social exclusion was associated with loneliness, depression, entrapment, and defeat (p < 0.05); the perceived burdensomeness was associated with none of the six psychological factors.

## Discussion

The aim of the present study was to examine the structure and validate the use of the Chinese version of the INQ-15 among a sample of TGW. Although the INQ has been widely translated and used in different countries to measure interpersonal needs and predict suicidal ideation, few studies have examined the psychometric properties of INQ in Chinese people and none of them focused on TGW despite their high suicide rates (Lai and Boag, 2021; Wang et al., 2021a). Considering the special social and cultural status in which TGW live (Winter et al., 2016), it is necessary to determine whether the INQ is a reliable and valid instrument for assessing interpersonal needs in this population.

Our results from the exploratory factor analysis supported the validity of a three-factor model. This model had six indicators for the perceived burdensomeness dimension, six indicators for the thwarted belongingness dimension, and three indicators for the social exclusion dimension. This was notably deviated from most previous findings that the INQ had only two dimensions of perceived burdensomeness and thwarted belongingness (Amini-Tehrani et al., 2021; Iliceto et al., 2021). However, a study investigating the construct validity of the INQ among clinically depressed and suicidal youth extracted a third factor from the TB factor and named it “perceived isolation” (Hunt et al., 2020). This three-factor solution including an additional factor “social isolation” for the INQ was confirmed later in another sample of Chinese migrant workers (Wang et al., 2021a). One commonality of these two validation studies is that they focus on a specific population who tend to have poor interpersonal bonds as a result of environmental or emotional maladjustment. Similarly, for the TGW who suffer from widespread discrimination and exclusion owing to their gender incongruence (White Hughto et al., 2015), interpersonal alienation, and social disconnection exist as severe psychosocial problems. Therefore, it is reasonable to recognize social exclusion, which represents the extent an individual feels alienated from society, as a third dimension in the measurement of interpersonal needs among TGW. This was confirmed by the IRT modeling and M2 test.

We further examine the psychometric properties of the Chinese version of the INQ after identifying the three-factor model. In the simultaneous regressions of the six observed variables on the three latent variables, thwarted belongingness was correlated with self-esteem and social support, and social exclusion was correlated with loneliness, depression, entrapment, and defeat, while perceived burdensomeness was correlated with none of these variables. These results supported the divergent validity of the INQ, which extended previous findings that the INQ had adequate convergent validity but not divergent validity in an important way (Van Orden et al., 2012; Silva et al., 2018). Van Orden suggested that the low divergent validity may be explained by the fact that belongingness is closely related to a wide range of psychological experiences (Van Orden et al., 2012). However, the present results showed that with the extraction of an additional factor, the different dimensions of the INQ could be clearly distinguished apart and social exclusion displayed stronger associations with those psychological measures than the other two dimensions. Although perceived burdensomeness was correlated with none of these variables in the present study, this may not indicate poor psychometric properties; rather, perceived burdensomeness, which is characterized by the feeling of self-hatred and being like a burden to others, is related to other distinct factors including competence, autonomy, and responsibility to family (Van Orden et al., 2012). Further study is needed to identify constructs that are related to perceived burdensomeness and clarify the convergent validity for this factor.

There are at least 400,000 transgender people in China based on the reported gender-affirming surgery cases (Jiang et al., 2014). However, the socio-cultural environment is still hostile for transgender people in China, which expresses through a high level of discrimination and a lack of acceptance and protection (Peng et al., 2019). Transgender people bear a greater burden of poor mental health, among which TGW are at the highest risk of suicidal ideation and suicidal attempt (Chen et al., 2019). However, studies on this vulnerable population with regard to suicide risk are still limited in China (Chen et al., 2019). The present study validated among a sample of TGW in China the use of the INQ to measure perceived unmet interpersonal needs, which have been identified as the proximal causes of suicidal thoughts and behaviors and indicate the presence of suicidal ideation (Joiner, 2005; Van Orden et al., 2008). The INQ has the potential to be a valuable instrument for detecting suicide risks because individuals are more likely to accurately report their thoughts on the INQ in contrast to the direct suicide assessment, given their fear of involuntary hospitalization or feelings of shame (Van Orden et al., 2012). Therefore, future studies can utilize this scale to further explore the suicidal ideation and attempt in Chinese TGW, which may provide important evidence in the development of effective suicide screening programs and prevention interventions in this population.

There are several study limitations that should be acknowledged. First, the sample size was relatively small owing to the scarcity of TGW, thus we could not divide the final sample into two groups to conduct exploratory factor analysis and CFA separately. However, the present findings provide an insight into the mental health problems of this hard-to-reach population. Second, the cross-sectional nature of the present study limited the evaluation of the psychometric properties of the INQ. To further validate the use of the Chinese version of the INQ in TGW, additional longitudinal studies are needed to explore the test–retest reliability and predictive validity of this scale in TGW. Third, as the present sample was limited to TGW in Shenyang, we should be cautious when generalizing the results to countrywide conclusions.

## Conclusion

The INQ has good psychometric properties with three distinct but related dimensions of interpersonal needs among TGW: perceived burdensomeness, thwarted belongingness, and social exclusion. To our knowledge, this is the first study that validates the use of the Chinese version of the INQ in a sample of TGW, which may provide important implication for future study. First, TGW experience a high level of perceived social exclusion which is closely associated with unmet interpersonal needs, thus additional strategies should be developed to reduce discrimination and improve the perception of fitting in for TGW. Second, future researchers and clinicians can use the Chinese version of the INQ as a reliable and valid assessment tool to further study the perceived interpersonal needs and better assess the suicide risks among TGW.

## Data Availability Statement

The raw data supporting the conclusions of this article will be made available by the authors, without undue reservation.

## Ethics Statement

The studies involving human participants were reviewed and approved by the Institutional Review Board of the School of Public Health, Shanghai Jiao Tong University School of Medicine. The patients/participants provided their written informed consent to participate in this study.

## Author Contributions

YC, HW, RC, YW, and LJ: study design. HW, YC, and RC: data collection. CX, XY, SL, LJ, and HC: data curation. YL, RW, and YC: writing—original draft and formal analysis. YC, YW, LJ, and LX: writing—review and editing. All authors have read and agreed to the published version of the manuscript.

## Funding

This work was supported by the National Natural Science Funds of China under Grant 71673187; the Shanghai Three-Year Action Plan for Public Health under Grants GWV-10.2-XD13, GWV-10.1-XK15, and GWV-10.1-XK18; the Strategic collaborative innovation team under Grant SSMU-ZLCX20180601; and the National Key Research and Development Project under Grants 2018YFC1705100 and 2018YFC1705103. The funding body had no role in the study design, collection, analysis, or interpretation of the data, writing the manuscript, or the decision to submit the paper for publication.

## Conflict of Interest

The authors declare that the research was conducted in the absence of any commercial or financial relationships that could be construed as a potential conflict of interest.

## Publisher’s Note

All claims expressed in this article are solely those of the authors and do not necessarily represent those of their affiliated organizations, or those of the publisher, the editors and the reviewers. Any product that may be evaluated in this article, or claim that may be made by its manufacturer, is not guaranteed or endorsed by the publisher.
