# Supplementary material for: Perceived Burdensomeness, Thwarted Belongingness, and Social Exclusion in Transgender Women: Psychometric Properties of the Interpersonal Needs Questionnaire
Source: Front Psychol. 2022 Feb 10;13:787809. doi: 10.3389/fpsyg.2022.787809 (PMC8868044; doi:10.3389/fpsyg.2022.787809)
Supplement: Supplementary file 1 [file Table_1.DOCX]

Table A1 Results of Monte Carlo simulation

|  | Estimates | | | S.E. Average | 95% Coverage |
| --- | --- | --- | --- | --- | --- |
|  | Population | Average | Std.Dev |  |  |
| F1 BY |  |  |  |  |  |
| A1 | 0.686 | 0.6835 | 0.0674 | 0.0659 | 0.944 |
| A2 | 0.730 | 0.7266 | 0.0653 | 0.0645 | 0.950 |
| A3 | 0.614 | 0.6119 | 0.0676 | 0.0679 | 0.951 |
| A4 | 0.800 | 0.7972 | 0.0619 | 0.0622 | 0.949 |
| A5 | 0.694 | 0.6910 | 0.0664 | 0.0656 | 0.945 |
| A6 | 0.657 | 0.6555 | 0.0671 | 0.0668 | 0.947 |
| F2 BY |  |  |  |  |  |
| A7 | 0.605 | 0.6037 | 0.0714 | 0.0701 | 0.948 |
| A8 | 0.578 | 0.5759 | 0.0709 | 0.0707 | 0.949 |
| A10 | 0.662 | 0.6594 | 0.0690 | 0.0685 | 0.947 |
| A13 | 0.672 | 0.6696 | 0.0691 | 0.0684 | 0.948 |
| A14 | 0.748 | 0.7456 | 0.0668 | 0.0662 | 0.948 |
| A15 | 0.615 | 0.6125 | 0.0701 | 0.0698 | 0.948 |
| F3 BY |  |  |  |  |  |
| A9 | 0.442 | 0.4401 | 0.0780 | 0.0770 | 0.947 |
| A11 | 0.714 | 0.7115 | 0.0743 | 0.0733 | 0.947 |
| A12 | 0.742 | 0.7398 | 0.0746 | 0.0733 | 0.946 |
| F1 WITH |  |  |  |  |  |
| F2 | 0.350 | 0.3513 | 0.0768 | 0.0758 | 0.944 |
| F3 | 0.648 | 0.6474 | 0.0642 | 0.0636 | 0.947 |
| F2 WITH |  |  |  |  |  |
| F3 | 0.472 | 0.4715 | 0.0798 | 0.0781 | 0.940 |

Std.Dev = standard deviation; S.E. Average = standard error average
